# Guidelines for reporting non-randomised pilot and feasibility studies

**DOI:** 10.1186/s40814-019-0499-1

**Published:** 2019-10-06

**Authors:** Gillian A. Lancaster, Lehana Thabane

**Affiliations:** School of Primary, Social and Community Care, Keele University, Newcastle-under-Lyme, UK

## Abstract

As the number of submissions to *Pilot and Feasibility Studies* increases, there is a need for good quality reporting guidelines to help researchers tailor their reports in a way that is consistent and helpful to other readers. The publication in 2016 of the CONSORT extension to pilot and feasibility trials filled a much-needed gap, but there still remains some uncertainty as to how to report pilot and feasibility studies that are not randomised. This editorial aims to provide some general guidance on how to report the most common types of non-randomised pilot and feasibility studies that are submitted to the journal. We recommend using the CONSORT extension to pilot and feasibility trials as the main reference document—it includes detailed elaboration and explanation of each item, and in most cases, simple adaptation, or non-use of items that are not applicable, will suffice. Several checklists found on the Equator website may provide helpful supplementary guidance, when used alongside the CONSORT extension, and we give some examples.

## Introduction

Since the inception of the BMC journal *Pilot and Feasibility Studies* in 2015 [1], the number of published studies has risen sharply each year, totalling 379 by the end of 2018. In 2016, the Consolidated Standards of Reporting Trials (CONSORT) extension to randomised pilot and feasibility trials and two related methodology papers were published by the Pilot and Feasibility Studies (PAFS) Working Group (see the “Acknowledgements” section) to aid researchers in the planning and reporting of these types of studies [2–5]. An associated PAFS website was created as a point of reference for information about pilot and feasibility studies and associated events (https://pilotandfeasibilitystudies.qmul.ac.uk/). Recently, we also published an editorial guide to the reporting of protocols of randomised pilot and feasibility trials [6], recommending the use of the CONSORT extension guideline [2, 3] alongside the SPIRIT (Standard Protocol Items: Recommendations for Interventional Trials) checklist [7].

These publications have focused on guidelines for reporting *randomised* pilot and feasibility trials, but as the number of manuscript submissions to the journal continues to increase (by 200% from 2015 to 2018), there has arisen a need for some guidance on the reporting of non-randomised pilot and feasibility studies. Many non-randomised studies are undertaken before a randomised pilot or feasibility trial takes place and may comprise a wide spectrum of study designs. In this editorial, we discuss the most common types of non-randomised studies seen in the journal and give guidance on how they should be reported. In most cases, we recommend referring to the CONSORT extension to pilot and feasibility trials [2, 3] as many of the items (excluding items that are specific to the randomisation nature of the study) will be relevant for reporting other types of pilot and feasibility studies, and the guideline provides helpful examples and commentary for each of the 26 items. Many other guidelines exist on the Equator (Enhancing the QUAlity and Transparency Of health Research) website (http://www.equator-network.org/), and with minor amendments, some can be adapted for reporting certain types of non-randomised studies.

This editorial is based on our experience of submissions to the journal over the past 4 years, and while it does not provide comprehensive coverage of all types of non-randomised pilot and feasibility studies, it will hopefully provide some useful suggestions and signposts to relevant guidance and examples as an aid to reporting these studies. A point to note here is that our work to date has shown that there is a lack of consensus over the usage of the words ‘pilot’ and ‘feasibility’ [4], and so as a consequence, both terms are currently being used interchangeably in the journal.

## Guidance for reporting non-randomised pilot and feasibility studies for submission to the journal

In the journal *Pilot and Feasibility Studies*, aided by the CONSORT extension to pilot and feasibility trials, authors are encouraged to report the purpose of a feasibility or pilot study in the context of the planned future study. Many types of non-randomised feasibility studies are at an earlier stage of preparation to that of a randomised pilot or feasibility trial. The proposed methodology and procedures for the main randomised controlled trial (RCT) may still be under development and not yet ready to fully pilot test. These studies usually focus on one or more related but substantive areas of development along the RCT preparatory pathway (e.g. intervention development, development of patient-reported outcome measures (PROMS), piloting of several components of the main trial and piloting the feasibility of implementation).

Moreover, not all pilot and feasibility studies relate to trials or interventional studies; some concern testing out design features of future large-scale cohort studies, such as the feasibility of roll-out across a wide area or being able to obtain buy-in from different stakeholders. Other researchers may want to test the feasibility of preliminary hypotheses of associations between variables that may be important to inform future research before any kind of intervention is developed or future study planned.

Table 1 lists the main types of non-randomised feasibility studies seen in the journal, and we provide guidance and examples for reference.

**Table 1:** Main types of non-randomised feasibility studies submitted to the journal, where to find guidance and published examples

Type of study	Equator website checklists and other helpful guidance	Published examples
Intervention development	TIDieR http://www.equator-network.org/reporting-guidelines/tidier/ Maximising the impact of qualitative research in feasibility studies for randomised controlled trials: guidance for researchers (O’Cathain et al): https://pilotfeasibilitystudies.biomedcentral.com/articles/10.1186/s40814-015-0026-y	Thematic series on intervention development available at: https://www.biomedcentral.com/collections/interventiondevelopment
Patient-Reported Outcome Measures (PROMS) development	CONSORT PRO (adapt alongside CONSORT extension to pilot trials) http://www.equator-network.org/reporting-guidelines/consort-pro/ COSMIN User Manual (comprehensive reference, useful risk of bias tool) https://cosmin.nl/wp-content/uploads/COSMIN-syst-review-for-PROMs-manual_version-1_feb-2018.pdf	Thematic series on pilot and feasibility testing of patient-reported outcome measures available at: https://www.biomedcentral.com/collections/pilotfeasibilityPROMs
Piloting several components of the trial	CONSORT extension to pilot trials (ignoring items not applicable) http://www.equator-network.org/reporting-guidelines/consort-2010-statement-extension-to-randomised-pilot-and-feasibility-trials/	Aging, Community and Health—Community Partnership Program before-after study [25]: https://pilotfeasibilitystudies.biomedcentral.com/articles/10.1186/s40814-016-0063-1 POWeR-RN non-randomised study with wait-list control [26] https://pilotfeasibilitystudies.biomedcentral.com/articles/10.1186/s40814-017-0122-2#Sec16
Implementation of research findings	CONSORT extension to pilot trials (ignoring items not applicable) http://www.equator-network.org/reporting-guidelines/consort-2010-statement-extension-to-randomised-pilot-and-feasibility-trials/ RE-AIM (Reach, Effectiveness, Adoption, Implementation, Maintenance) framework for evaluating interventions http://www.re-aim.org/ Please note that when applying RE-AIM to pilot and feasibility studies, ‘potential effectiveness’ only should be addressed.	Thematic series on implementation science and practice forthcoming at: https://www.biomedcentral.com/collections/implementationscience-pilotstudies GLA:D® Back before-after study [28]: https://pilotfeasibilitystudies.biomedcentral.com/articles/10.1186/s40814-019-0448-z GenerationPMTO before-after study [29] https://pilotfeasibilitystudies.biomedcentral.com/articles/10.1186/s40814-019-0476-8
Feasibility studies in preparation for a cohort or other large scale study	STROBE (ignoring items not applicable) http://www.equator-network.org/reporting-guidelines/strobe/ CONSORT extension to pilot trials (ignoring items not applicable) http://www.equator-network.org/reporting-guidelines/consort-2010-statement-extension-to-randomised-pilot-and-feasibility-trials/ • Ensure there is adequate explanation as to why the study is a feasibility study, and state clear feasibility objectives • Ensure a formal sample size calculation is reported if hypothesis testing is carried out	Community-based paediatric respiratory infection surveillance cohort study [31]: https://pilotfeasibilitystudies.biomedcentral.com/articles/10.1186/s40814-018-0371-8 Prognosis of patients with apparent treatment-resistant hypertension [32]: https://pilotfeasibilitystudies.biomedcentral.com/articles/10.1186/s40814-018-0232-5
Feasibility studies that test preliminary hypotheses of association	STROBE (ignoring items not applicable) http://www.equator-network.org/reporting-guidelines/strobe/ CONSORT extension to pilot trials (ignoring items not applicable) http://www.equator-network.org/reporting-guidelines/consort-2010-statement-extension-to-randomised-pilot-and-feasibility-trials/ • Ensure there is adequate explanation as to why the study is a feasibility study, and state clear feasibility objectives • Ensure a formal sample size calculation is reported if hypothesis testing is carried out	Is cognitive function in delirium associated with EEG frequency band connectivity (case-control study) [33]? https://pilotfeasibilitystudies.biomedcentral.com/articles/10.1186/s40814-018-0388-z Are foetus mouth movements associated with sound stimulation in the womb [34]? https://pilotfeasibilitystudies.biomedcentral.com/articles/10.1186/s40814-016-0053-3

### Intervention development

Studies that describe intervention development typically adopt mainly qualitative methods. The Template for Intervention Description and Replication (TIDieR) guideline exists for reporting intervention description [8]. Intervention development studies often describe a theoretical model that underpins the reasoning behind the intervention and through literature review or focus group work develop a feasible intervention model. This new intervention is then tried out on a small number of patients and adopted or modified as necessary.

The first thematic series of the journal covered intervention development and drew upon the expertise of guest editor, Professor Pat Hoddinott, to oversee the papers contributing to the series. Around the same time, Professor Alicia O’Cathain and colleagues published a guidance paper on maximising the impact of qualitative research in feasibility studies for RCTs (a highly accessed article) [9]. Hand in hand with the nine papers in the thematic series, these provide a good set of examples covering issues of complex intervention development [10–12] and strategic optimisation [13], a person-based approach to enhancing acceptability [14], intervention mapping [15] and obtaining clinical collaboration through a Knowledge to Action framework [16].

### Development of PROMs

PROM development, or development of any questionnaire-based outcome measure, has some methodological similarities to the previous section in terms of selection of the proposed items for the PROM. Items generally stem from an underlying theoretical model and literature review, aided by focus group work with some preliminary testing. The PROM is then assessed for its preliminary reliability and validity in certain patient populations related to its intended use. In our second thematic series of the journal, guest editor, Professor Georgina Jones, presents seven papers that represent the types of pilot work that might take place in PROM development, including issues of translation and back-translation for use in another language [17], time and cost of administration [18], technology-based assessment [19] and the use of e-PROMS [20]. In another study, the authors follow the RE-AIM (Reach, Effectiveness, Adoption, Implementation, Maintenance) framework [21] to pilot and evaluate use at clinic of an adolescent needs assessment tool for type 1 diabetes [22].

The CONSORT Patient-Reported Outcomes (PRO) guideline for the reporting of PROMs in main RCTs [23] may provide some further help but it should be adapted in line with the CONSORT extension to pilot and feasibility trials. The COSMIN (COnsensus-based Standards for the selection of health Measurement INstruments) guideline for systematic reviews of PROMs [24] is a comprehensive document to also be aware of especially when reporting aspects of preliminary reliability and validity and when considering the design of a future large-scale validation study.

### Piloting several components of the trial

Quite often enough may be known about the study design (e.g. from conducting previous trials in the same area) to not warrant a fully randomised pilot or feasibility trial. But it may still be necessary to try out certain aspects of the intervention delivery to ensure it will work. Generally, either a before-after study design testing out processes related to the intervention arm only, or processes related to the delivery of both arms without randomisation will suffice. In these cases, we would still recommend using the CONSORT extension to pilot and feasibility trials, as it can usually be readily adapted to these situations. Any items not applicable, for example, items 8a–10 about randomisation, can be ignored in a before-after single-arm study or adapted to non-random allocation for a two-arm non-randomised study.

One example of a before-after study examines the feasibility of the Aging, Community and Health—Community Partnership Program, an inter-professional, nurse-led programme to promote diabetes self-management in older adults with type 2 diabetes and multiple chronic conditions [25]. A non-randomised study example adopts the RE-AIM framework [21] to assess the feasibility of implementing a modified weight loss programme, Positive Online Weight Reduction for Royal Navy (POWeR-RN), in overweight and obese navy personnel with a wait-list control group [26].

### Piloting the feasibility of implementation of research findings

Implementation of methods to promote the systematic uptake of research findings, including interventions and other evidence-based practices into routine practice, is the topic of our third thematic series—currently an open call. Piloting plans for future implementation and evaluation of research programmes and showing them to be feasible is an important part of implementation research on the continuum of getting research into current practice. While there are journals focussing on implementation research, the preparation that goes into these programmes is not always apparent or well-reported.

Again, we would recommend using the CONSORT extension to pilot and feasibility trials as the basis for reporting such studies with suitable adaptation of items where necessary. The one published paper from the call to date describes the implementation into nutritional rehabilitation units in Malawi of the Kusamala Program, an interactive counselling programme for primary caregivers of children with severe acute malnutrition [27]. In the GLA:D® Back (Good Life with osteoArthritis in Denmark) before-after study, physiotherapists and chiropractors were trained in intervention delivery of standardised care following national guidelines for low back pain to plan a future implementation-effectiveness study [28]. Another example seeks to improve the implementation of evidence-based practices by teaching the Generation Parent Management Training Oregon (GenerationPMTO®) model, a parenting intervention, in a university graduate curriculum [29]. The RE-AIM framework has also been used in this context [21, 22].

### Feasibility studies in preparation for a cohort or other large scale study

While the majority of studies submitted to the journal are in preparation for a main future RCT, the journal is also open to submission of articles related to pilot and feasibility work for cohort studies or other large-scale observational studies. The STROBE (Strengthening the Reporting of Observational Studies in Epidemiology) guideline [30] provides a checklist of items that should be included in these types of reports, and most items are applicable to pilot and feasibility studies. However, care should be taken to state clearly the aims and feasibility objectives for the pilot work which should differ from those of the main future study. For this reason, it is recommended that the STROBE checklist is used alongside the CONSORT extension to pilot and feasibility trials to ensure that all items relate or are adapted to issues of feasibility.

Examples of feasibility cohort studies that have been published in the journal to date have concerned the feasibility of recruiting and following up children with respiratory tract infections in the community, including collecting microbiological, symptom severity and duration data [31], and determining the feasibility of recruiting practices and patients with apparent treatment-resistant hypertension for data collection and follow-up of outcomes [32].

### Feasibility studies that test preliminary hypotheses of association

Sometimes, it is necessary to test preliminary hypotheses of associations between variables which if found to be promising may lead to intervention development or other preliminary work. In other cases, the associations may be in preparation for a trial. These studies are in the minority in the journal, but there are several examples to draw upon. Some adopt observational study designs and some are non-randomised experiments. We again recommend the use of the STROBE guideline alongside the CONSORT extension to pilot and feasibility trials with suitable adaptation of items as necessary.

The two examples in Table 1 look at associations between delirium and electroencephalography (EEG) frequency band connectivity readings as potential future therapeutic and diagnostic biomarkers [33] and whether sound stimulation in the womb is associated with mouth movements in the foetus [34]. If these associations are observed, then further future research can be planned.

## Discussion

We have provided guidance for reporting non-randomised pilot and feasibility studies. In most cases, existing guidelines can be adapted and utilised for this purpose, and we have taken some sample guidelines from the Equator website. While we have categorised studies into several common types, as can be seen from the published examples, there is overlap in the types of studies discussed with some examples fitting under more than one sub-heading.

In this editorial, we have taken all examples from the journal *Pilot and Feasibility Studies*. Many journals still do not have a policy of publishing pilot and feasibility studies. In a previous review of four subject-specific journals and three general mainstream medical journals, Lancaster et al. [35] identified only 90/4449 (2%) research studies published between 2000 and 2001 that called themselves ‘pilot’ or ‘feasibility’ studies. The majority were studies piloting a new treatment or technique (70%), piloting guidelines (11%), or screening programmes (5%). Surprisingly at the time, only 4 out of the 90 pilot/feasibility studies across all 7 journals were identified as being in preparation for a future RCT. Today, with the publication of the CONSORT extension to pilot and feasibility trials in 2016 [2, 3] and aided by other influential papers [36, 37], this number has improved, and we are starting to see phases of pilot and feasibility work published along the RCT preparatory pathway.

Most research submitted to the journal reports on one substantive phase of work at a time, addressing intervention development work or uncertainties in the study design. Research protocol submissions may describe the substantive preparatory phases altogether in one publication as a set of planned sub-studies, for example, theoretical review, intervention development and testing (in a few patients), feasibility testing in a larger patient sample, feasibility of implementation into practice and acceptability to key stakeholders. Problems can arise when researchers attempt to report multiple results from each phase within one paper, and this poses a risk of underreporting all of the pertinent findings.

The publication and sharing of detailed feasibility work has many benefits for researchers across disciplines in learning from each other, in reusing techniques that have proved successful and in avoiding similar pitfalls. Much preparatory and exploratory work is linked to the development and evaluation of complex interventions and as such should comply with the UK Medical Research Council (MRC) guidance [38]. This guidance is currently being updated, and we welcome mention of the progress that has been made to date in providing a more comprehensive framework for reporting pilot and feasibility studies [2–4].

## Conclusion

We hope that this editorial will be helpful to researchers when reporting non-randomised feasibility and pilot studies. We recommend that authors use the current guidance available and ensure items are included to emphasise the goal of feasibility, such as specific feasibility objectives, feasibility outcomes and progression criteria. In writing this guidance, we have tried to identify and clarify the main kinds of issues we repeatedly see in our roles as Editors-in-Chief in an attempt to help researchers in reporting their work. We would like to end by re-iterating the message that reporting guideline publications that contain explanation and elaboration commentary on each item are very useful reference documents to consult not only at the end of a study when writing up the results, but also at the planning stage of a study when constructing an appropriate study design.

## Data Availability

Not applicable

## Abbreviations

CONSORT: Consolidated Standards of Reporting Trials
COSMIN: COnsensus-based Standards for the selection of health Measurement INstruments
EQUATOR: Enhancing the QUAlity and Transparency Of health Research
GenerationPMTO: Generation Parent Management Training Oregon Model
GLA:D®: Good Life with osteoArthritis in Denmark
MRC: Medical Research Council
PAFS: Pilot and Feasibility Studies
PRO: Patient-Reported Outcomes
PROM: Patient-reported outcome measure
POWeR-RN: Positive Online Weight Reduction for Royal Navy
RCT: Randomised controlled trial
RE-AIM: Reach, Effectiveness, Adoption, Implementation, Maintenance
SPIRIT: Standard Protocol Items: Recommendations for Interventional Trials
STROBE: Strengthening the Reporting of Observational Studies in Epidemiology
TIDieR: Template for Intervention Description and Replication

## References

[1] Lancaster GA. Pilot and feasibility studies come of age! *Pilot and Feasibility Studies* 2015;1,1. *Available at:* https://pilotfeasibilitystudies.biomedcentral.com/ (last accessed 2/7/19).

[2] Eldridge S, Chan C, Campbell M, Bond C, Hopewell S, Thabane L, Lancaster GA (2016). CONSORT Statement: extension to randomised pilot and feasibility trials. BMJ.

[3] Eldridge S, Chan C, Campbell M, Bond C, Hopewell S, Thabane L, Lancaster GA (2016). CONSORT Statement: extension to randomised pilot and feasibility trials. Pilot and Feasibility Studies.

[4] Eldridge S, Lancaster GA, Campbell M, Thabane L, Hopewell S, Coleman C, Bond C (2016). Defining feasibility and pilot studies in preparation for randomised controlled trials: using consensus methods and validation to develop a conceptual framework. PloS One.

[5] Thabane L, Hopewell S, Lancaster GA, Bond C, Coleman C, Campbell M, Eldridge S (2016). Methods and processes for development of a CONSORT extension for reporting pilot randomized controlled trials. Pilot and Feasibility Studies.

[6] Thabane L, Lancaster GA. A guide to the reporting of protocols of pilot and feasibility trials. *Pilot and Feasibility Studies*. 2019;5:37. 10.1186/s40814-019-0423-8 PMC6393983 30858987

[7] Chan A-W, Tetzlaff JM, Altman DG, Laupacis A, Gøtzsche PC, Krleža-Jerić K, Hróbjartsson A, Mann H, Dickersin K, Berlin J, Doré C, Parulekar W, Summerskill W, Groves T, Schulz K, Sox H, Rockhold FW, Rennie D, Moher D (2013). SPIRIT 2013 Statement: defining standard protocol items for clinical trials. Ann Intern Med..

[8] Hoffman T, Glasziou P, Boutron I, Milne R, Perera R, Moher D, Altman D, Barbour V, Macdonald H, Johnston M, Lamb S, Dixon-Woods M, McCulloch P, Wyatt J, Chan A, Michie S (2014). Better reporting of interventions: template for intervention description and replication (TIDieR) checklist and guide. BMJ..

[9] O’Cathain A, Hoddinott P, Lewin S, Thomas KJ, Young B, Adamson J, Jansen YJFM, Mills N, Moore G, Donovan JL. Maximising the impact of qualitative research in feasibility studies for randomised controlled trials: guidance for researchers. *Pilot and Feasibility Studies*. 2015;1:32. 10.1186/s40814-015-0026-y PMC5154038 27965810

[10] Sugg HVR, Richards DA, Frost J (2017). Optimising the acceptability and feasibility of novel complex interventions: an iterative, person-based approach to developing the UK Morita therapy outpatient protocol. Pilot and Feasibility Studies.

[11] Winder R, Richards SH, Campbell JL, Richards DA, Dickens C, Gandhi M, Wright C, Turner K (2017). Development and refinement of a complex intervention within cardiac rehabilitation services: experiences from the CADENCE feasibility study. Pilot and Feasibility Studies.

[12] Murray J, Williams B, Hoskins G, Skar S, McGhee J, Treweek S, Sniehotta FF, Sheikh A, Brown G, Hagen S, Cameron L, Jones C, Gauld D (2016). A theory-informed approach to developing visually mediated interventions to change behaviour using an asthma and physical activity intervention exemplar. Pilot and Feasibility Studies.

[13] Levati S, Campbell P, Frost R, Dougall N, Wells M, Donaldson C, Hagen S (2016). Optimisation of complex health interventions prior to a randomised controlled trial: a scoping review of strategies used. Pilot and Feasibility Studies.

[14] Yardley L, Ainsworth B, Arden-Close E, Muller I (2015). The person-based approach to enhancing the acceptability and feasibility of interventions. Pilot and Feasibility Studies.

[15] Greaves CJ, Wingham J, Deighan C, Doherty P, Elliott J, Armitage W, Clark M, Austin J, Abraham J, Frost J, Singh S, Jolly K, Paul K, Taylor L, Buckingham S, Davis R, Dalal H, Taylor RS (2016). Optimising self-care support for people with heart failure and their caregivers: development of the Rehabilitation Enablement in Chronic Heart Failure (REACH-HF) intervention using intervention mapping. Pilot and Feasibility Studies.

[16] Sturgiss EA, Douglas K (2016). A collaborative process for developing a weight management toolkit for general practitioners in Australia—an intervention development study using the Knowledge To Action framework. Pilot and Feasibility Studies.

[17] Schnohr CW, Rasmussen CL, Langberg H, Bjørner JB (2017). Danish translation of a physical function item bank from the Patient-Reported Outcome Measurement Information System (PROMIS). Pilot and Feasibility Studies.

[18] Skinner MW, Chai-Adisaksopha C, Curtis R, Frick N, Nichol M, Noone D, O’Mahony B, Page D, Stonebraker JS, Iorio A (2018). The Patient Reported Outcomes, Burdens and Experiences (PROBE) Project: development and evaluation of a questionnaire assessing patient reported outcomes in people with haemophilia. Pilot and Feasibility Studies.

[19] Khetani MA, McManus BM, Arestad K, Richardson Z, Charlifue-Smith R, Rosenberg C, Rigau B (2018). Technology-based functional assessment in early childhood intervention: a pilot study. Pilot and Feasibility Studies.

[20] O’Connell S, Palmer R, Withers K, Saha N, Puntoni S, Carolan-Rees G (2018). Requirements for the collection of electronic PROMS either “in clinic” or “at home” as part of the PROMs, PREMs and Effectiveness Programme (PPEP) in Wales: a feasibility study using a generic PROM tool. Pilot and Feasibility Studies.

[21] Glasgow RE, Vogt TM, Boles SM (1999). Evaluating the public health impact of health promotion interventions: the RE-AIM framework. Am J Public Health..

[22] Cooper H, Lancaster GA, Gichuru P, Peak M (2018). A mixed methods study to evaluate the feasibility of using the Adolescent Diabetes Needs Assessment Tool App in paediatric diabetes care in preparation for a longitudinal cohort study. Pilot and Feasibility Studies.

[23] Calvert M, Blazeby J, Altman DG, Revicki DA, Moher D, Brundage MD, CONSORT PRO Group (2013). Reporting of patient-reported outcomes in randomized trials: the CONSORT PRO extension. JAMA..

[24] Mokkink LB, Prinsen CAC, Patrick DL, Alonso J, Bouter LM, De Vet HCW, Terwee CB (2018). COSMIN methodology for systematic reviews of Patient-Reported Outcome Measures (PROMs) user manual.

[25] Markle-Reid M, Ploeg J, Fisher K, Reimer H, Kaasalainen S, Gafni A, Gruneir A, Kirkconnell R, Marzouk S, Akhtar-Danesh N, Thabane L, Rojas-Fernandez C, Upshur R (2016). The Aging, Community and Health Research Unit—Community Partnership Program for older adults with type 2 diabetes and multiple chronic conditions: a feasibility study. Pilot and Feasibility Studies.

[26] Garip G, Morton K, Bridger R, Yardley L (2017). Evaluating the feasibility of a web-based weight loss programme for naval service personnel with excess body weight. Pilot and Feasibility Studies.

[27] Daniel AI, van den Heuvel M, Gladstone M, Bwanali M, Voskuijl W, Bourdon C, Potani I, Fernandes S, Njirammadzi J, Bandsma RHJ (2018). A mixed methods feasibility study of the Kusamala Program at a nutritional rehabilitation unit in Malawi. Pilot and Feasibility Studies.

[28] Kongsted A, Hartvigsen J, Boyle E, Ris I, Kjaer P, Thomassen L, Vach W (2019). GLA:D® Back: group-based patient education integrated with exercises to support self-management of persistent back pain — feasibility of implementing standardised care by a course for clinicians. Pilot and Feasibility Studies.

[29] Baumann AA, Domenech Rodríguez MM, Wieling E, Parra-Cardona JR, Rains L, Forgatch M (2019). Teaching GenerationPMTO, an evidence-based parent intervention, in a university setting using a blended learning strategy. Pilot and Feasibility Studies.

[30] Elm Erik von, Altman Douglas G, Egger Matthias, Pocock Stuart J, Gøtzsche Peter C, Vandenbroucke Jan P (2007). Strengthening the reporting of observational studies in epidemiology (STROBE) statement: guidelines for reporting observational studies. BMJ.

[31] Anderson EC, Ingle S, Muir M, Beck CR, Leeming JP, Kesten J, Cabral C, Hay AD (2018). Population-based paediatric respiratory infection surveillance: a prospective inception feasibility cohort study. Pilot and Feasibility Studies.

[32] Hayes P, Kielty H, Casey M, Glynn LG, Molloy GJ, Durand H, Newell J, Murphy AW (2018). Prognosis of patients with apparent treatment-resistant hypertension—a feasibility study. Pilot and Feasibility Studies.

[33] Fleischmann R, Traenkner S, Kraft A, Schmidt S, Schreiber SJ, Brandt SA (2019). Delirium is associated with frequency band specific dysconnectivity in intrinsic connectivity networks: preliminary evidence from a large retrospective pilot case-control study. Pilot and Feasibility Studies.

[34] Reissland N, Francis B, Buttanshaw L, Austen JM, Reid V. Do fetuses move their lips to the sound that they hear? An observational feasibility study on auditory stimulation in the womb. *Pilot and Feasibility Studies*. 2016;2:14. 10.1186/s40814-016-0053-3 PMC5154120 27965834

[35] Lancaster GA, Dodd SR, Williamson PR (2004). Design and analysis of pilot studies: recommendations for good practice. Journal of Evaluation in Clinical Practice.

[36] Thabane L, Ma J, Chu R, Cheng J, Ismaila A, Rios LP, Robson R, Thabane M, Goldsmith CH. A tutorial on pilot studies: the what, why and how. *BMC Medical Research Methodology*. 2010;**10**(1). 10.1186/1471-2288-10-1 PMC2824145 20053272

[37] Arain M, Campbell MC, Cooper CL, Lancaster GA. What is a pilot or feasibility study? A review of current practice and editorial policy. *BMC Medical Research Methodology*. 2010;10:67. 10.1186/1471-2288-10-67 PMC2912920 20637084

[38] Craig P, Dieppe P, Macintyre S, Mitchie S, Nazareth I, Petticrew M (2008). Developing and evaluating complex interventions: the new Medical Research Council guidance. BMJ.

